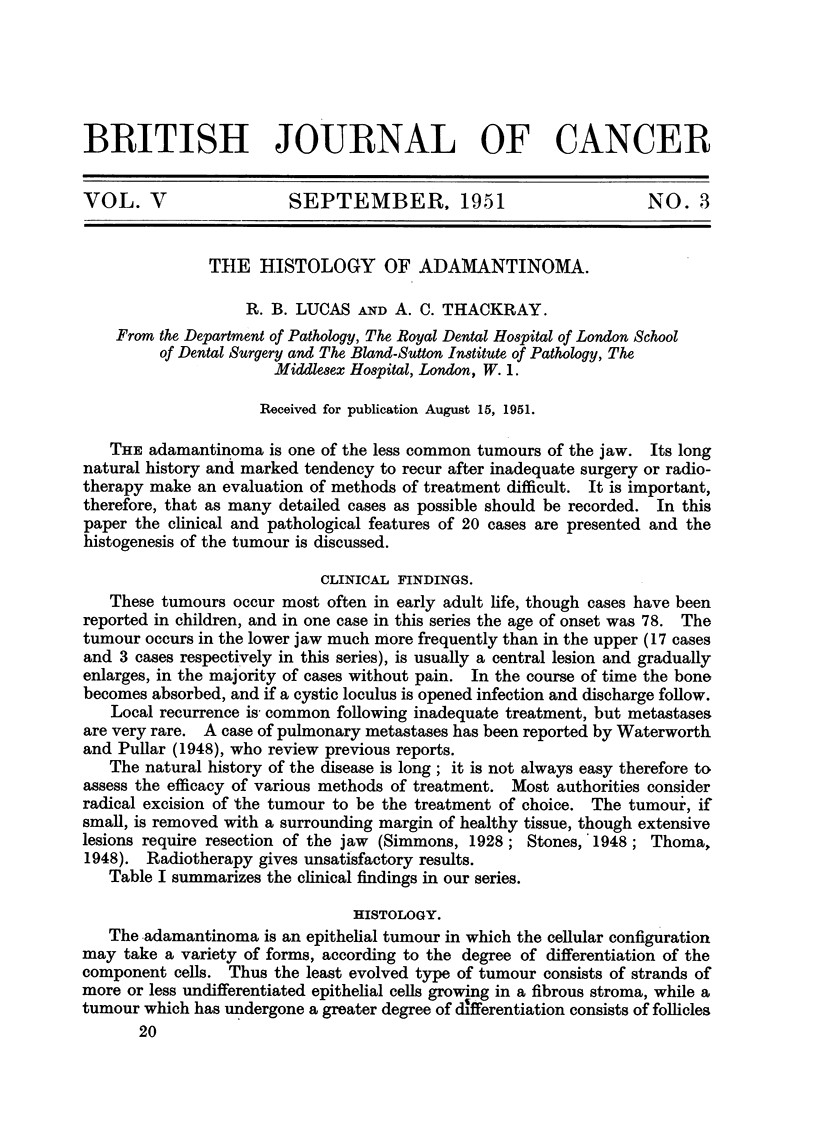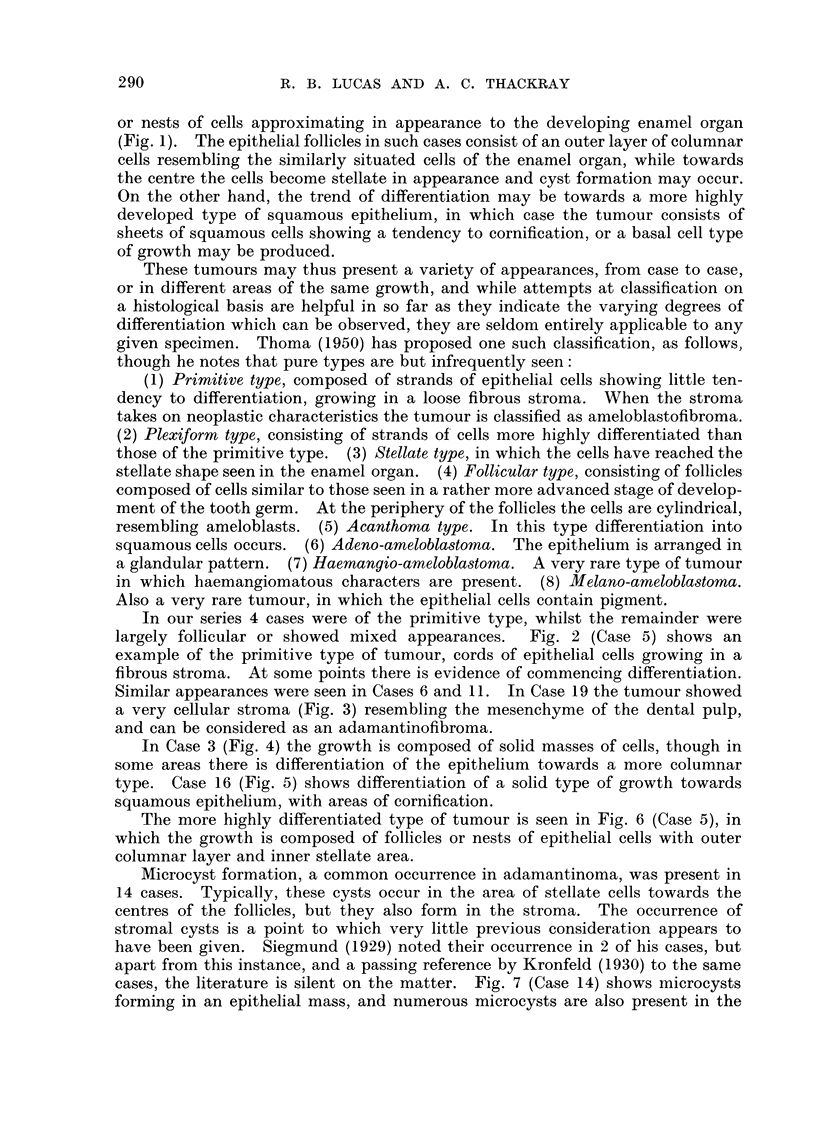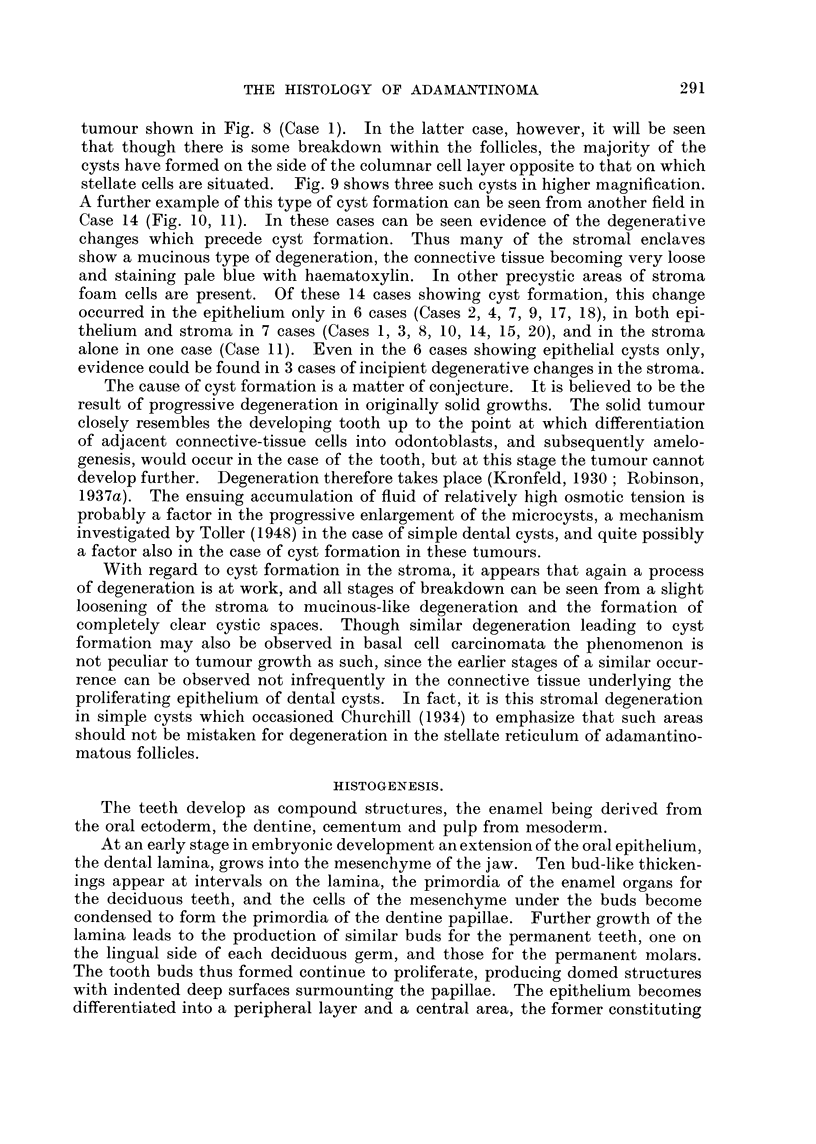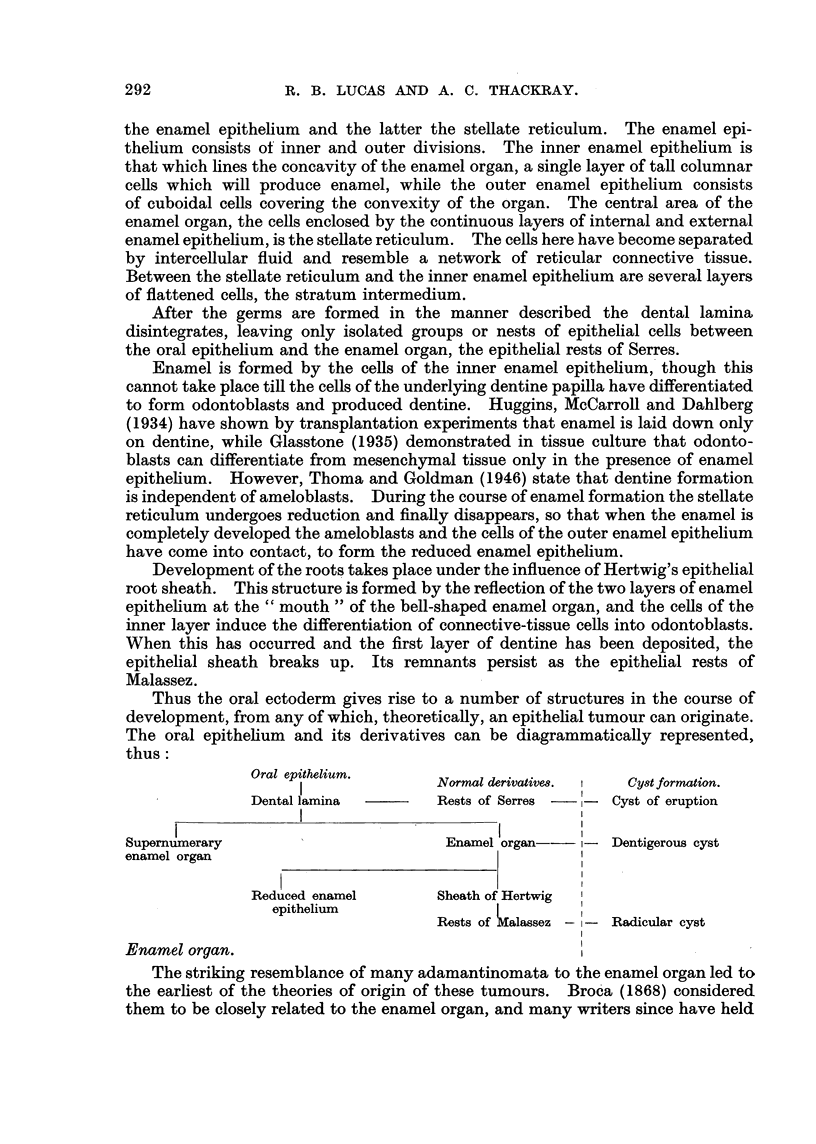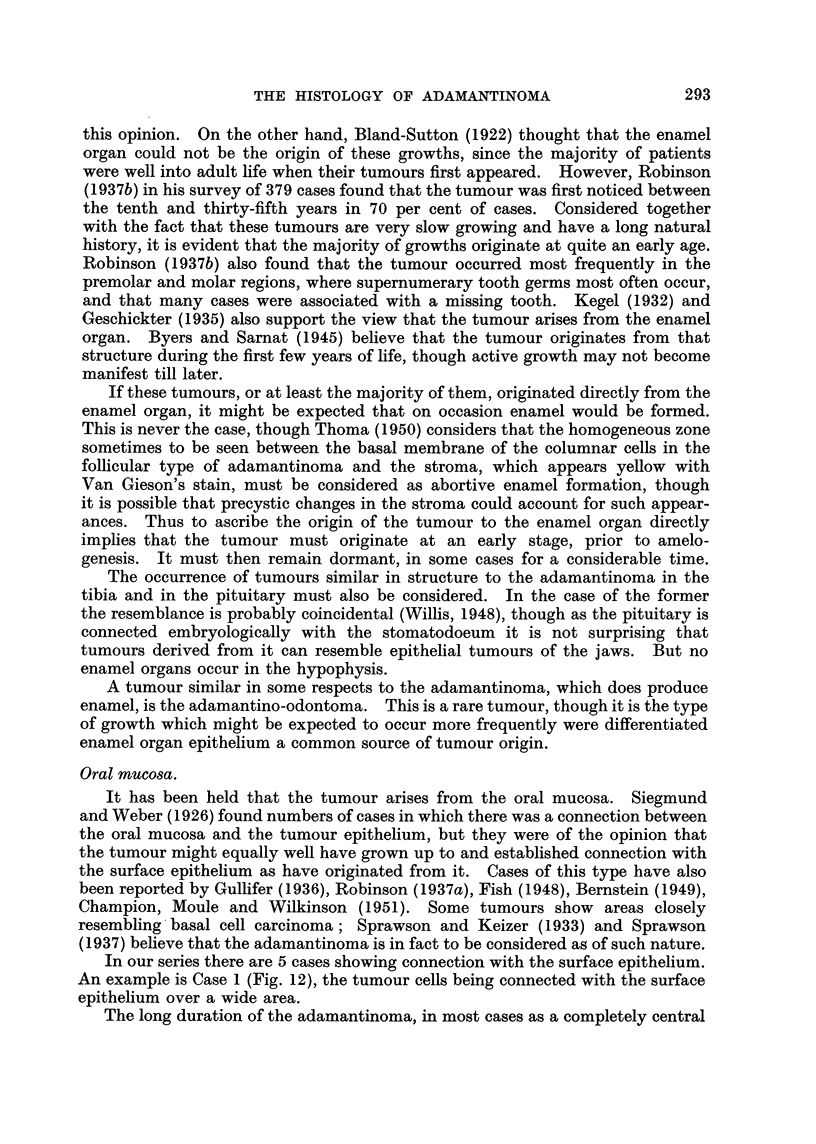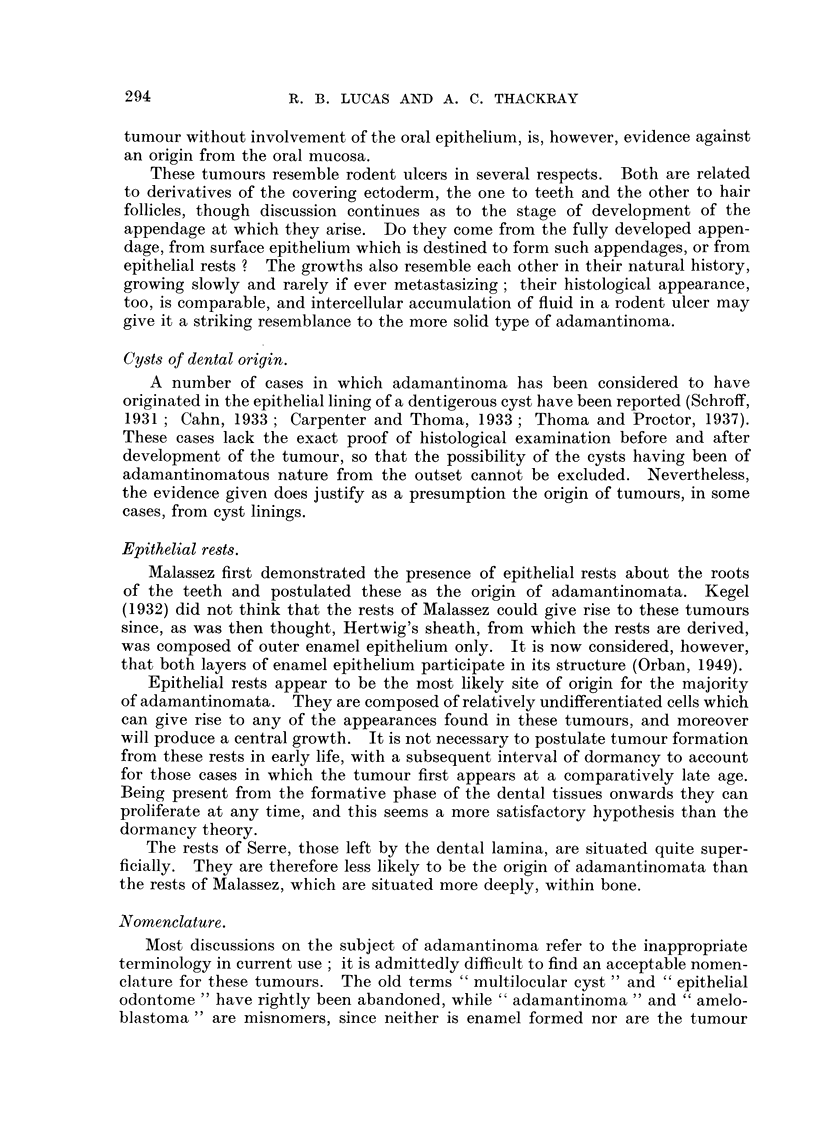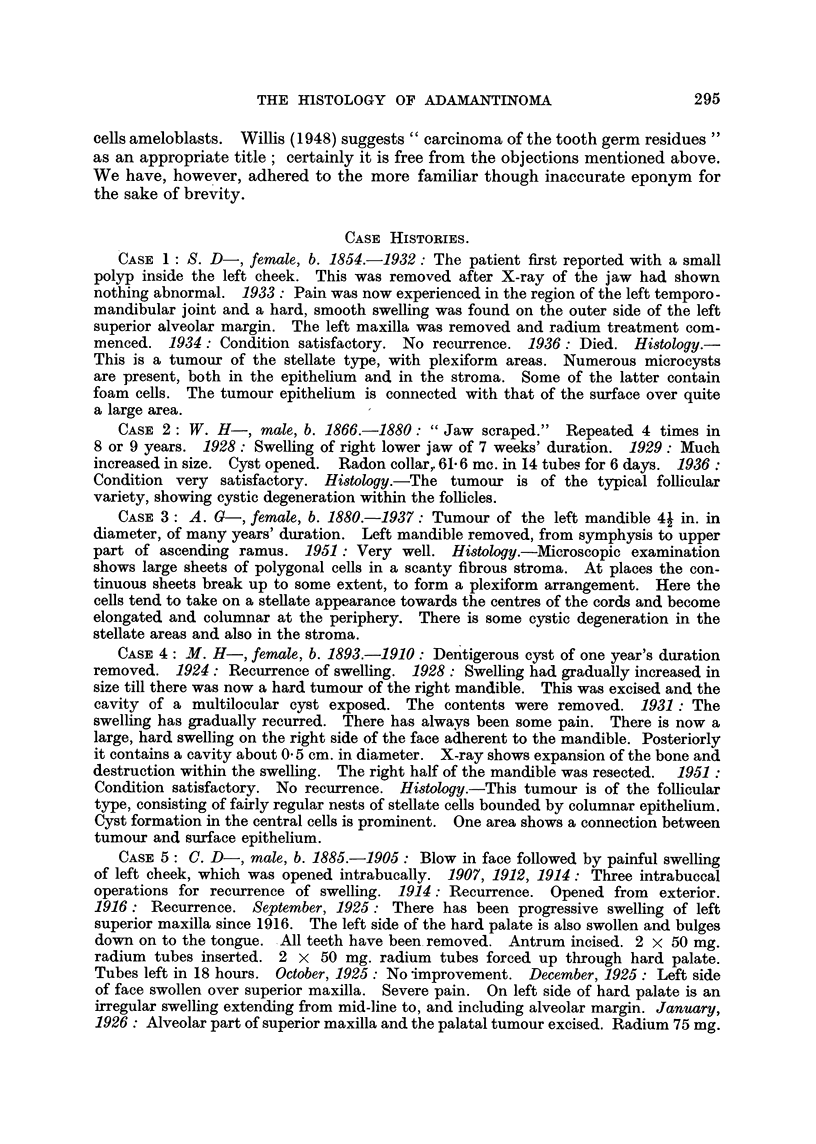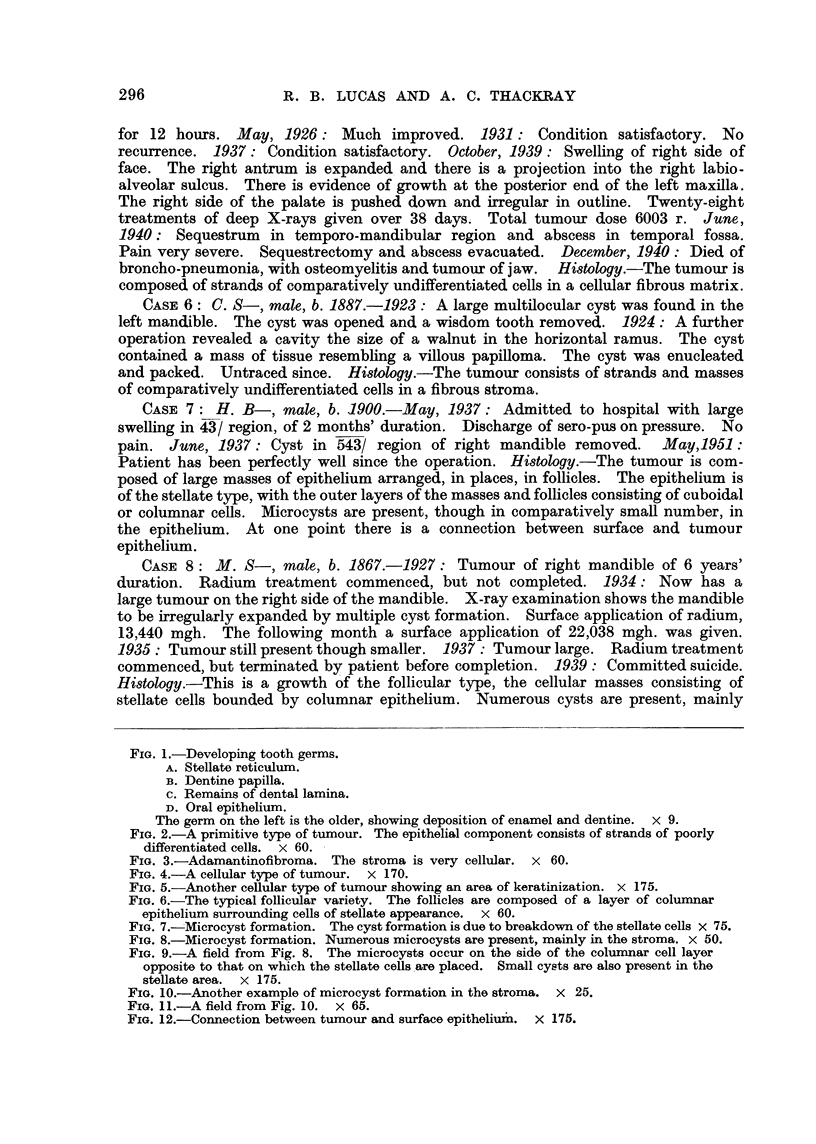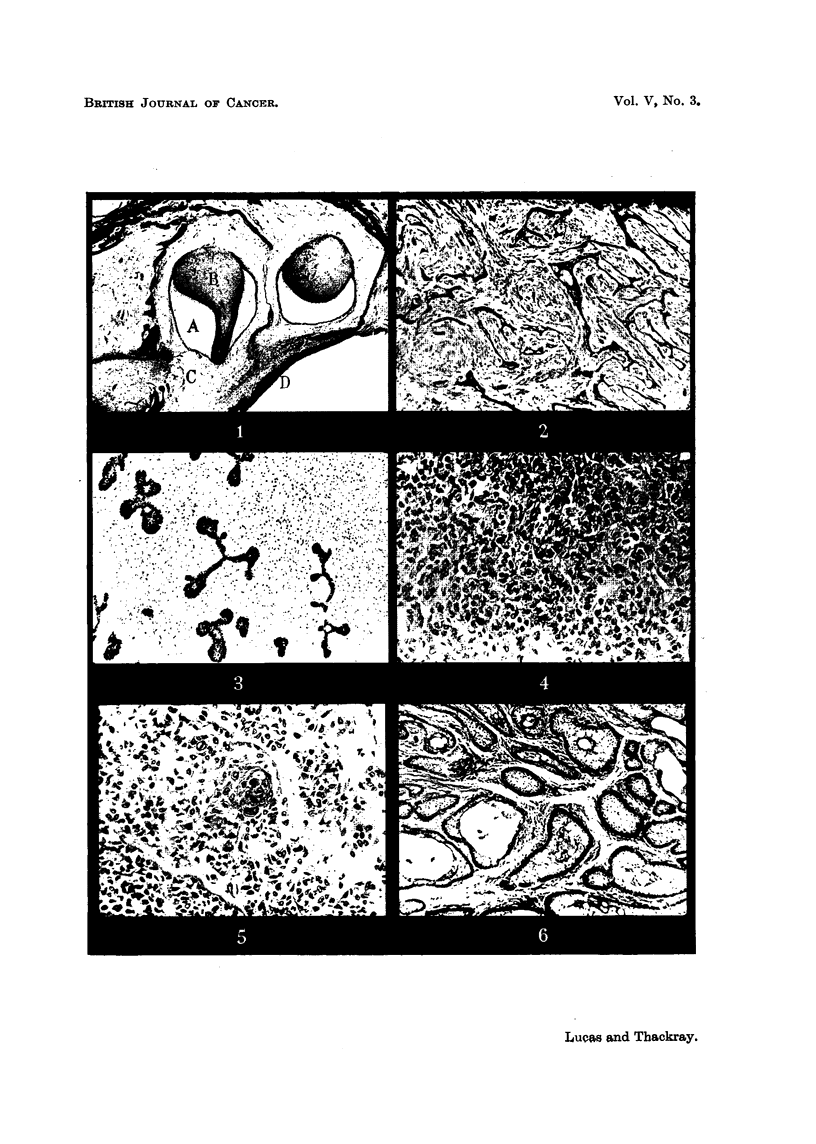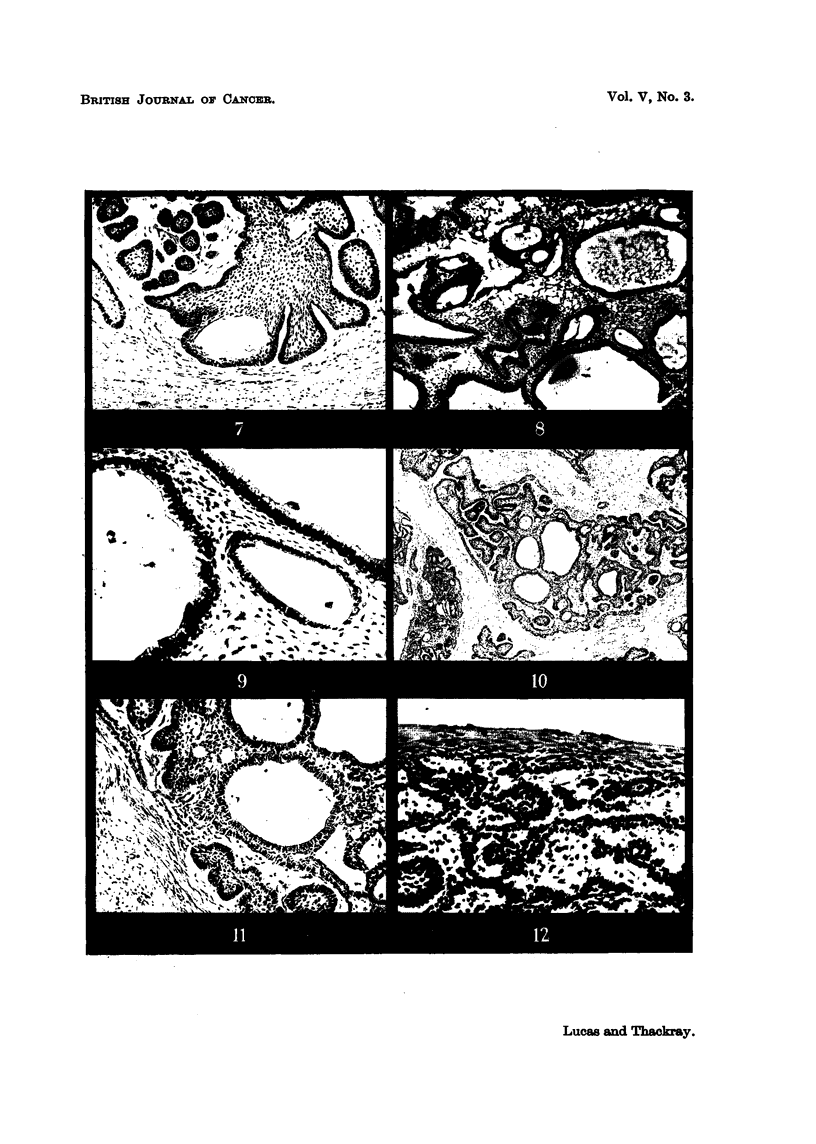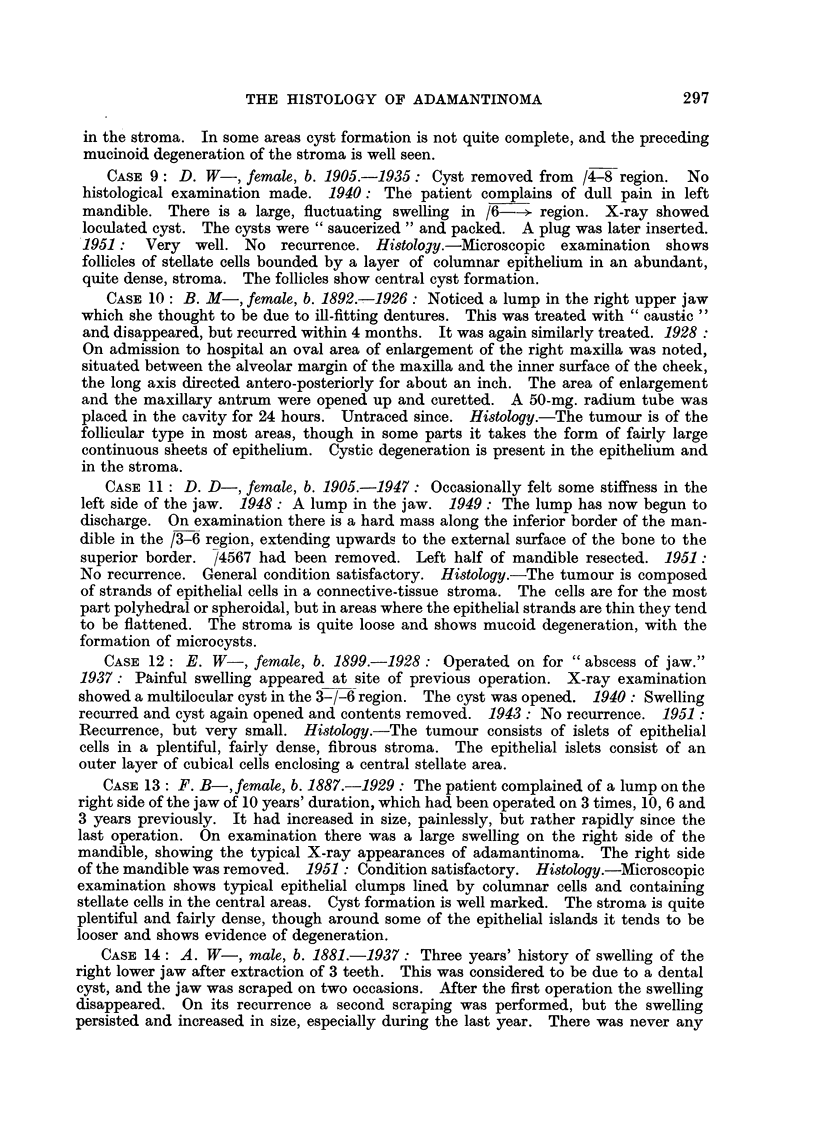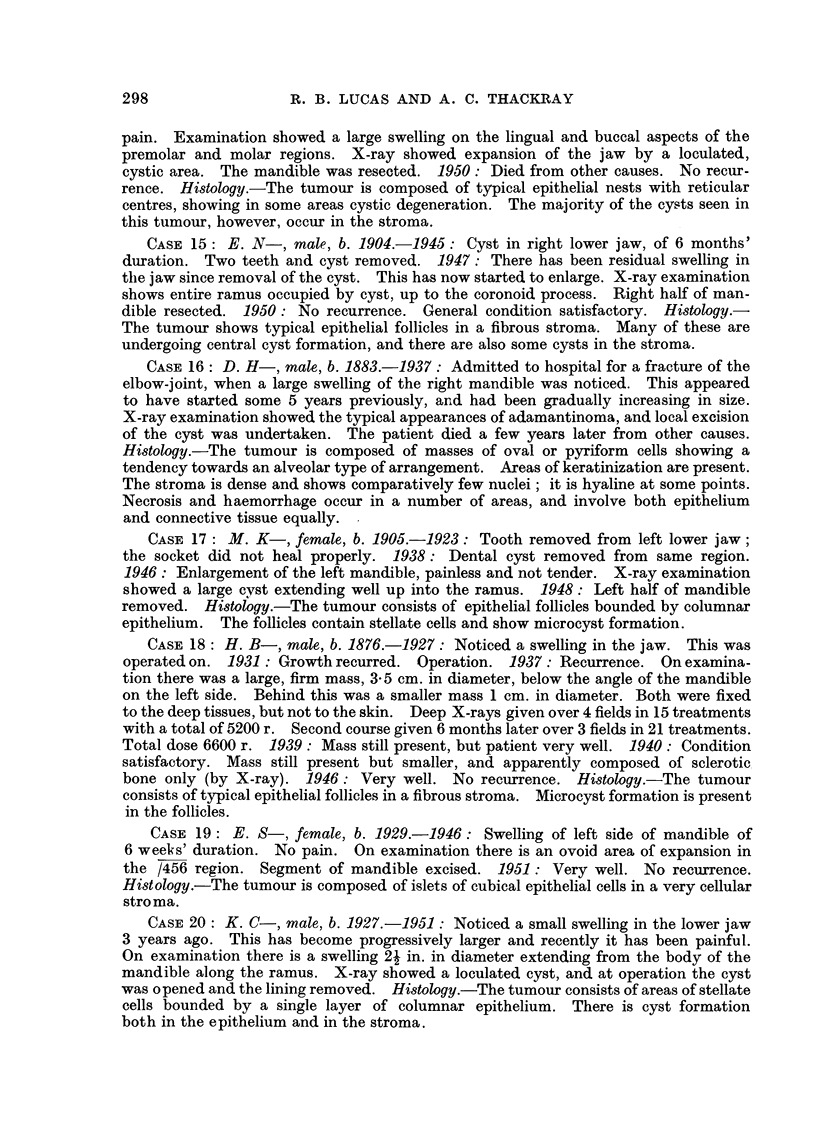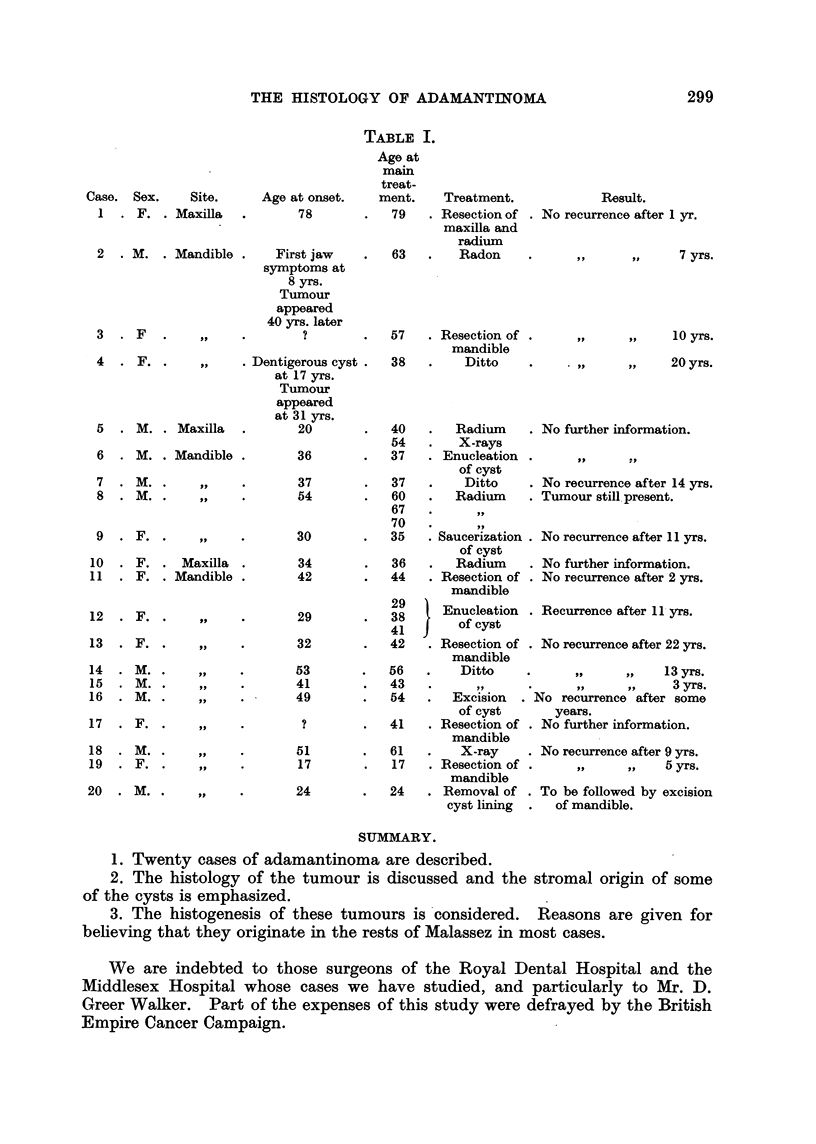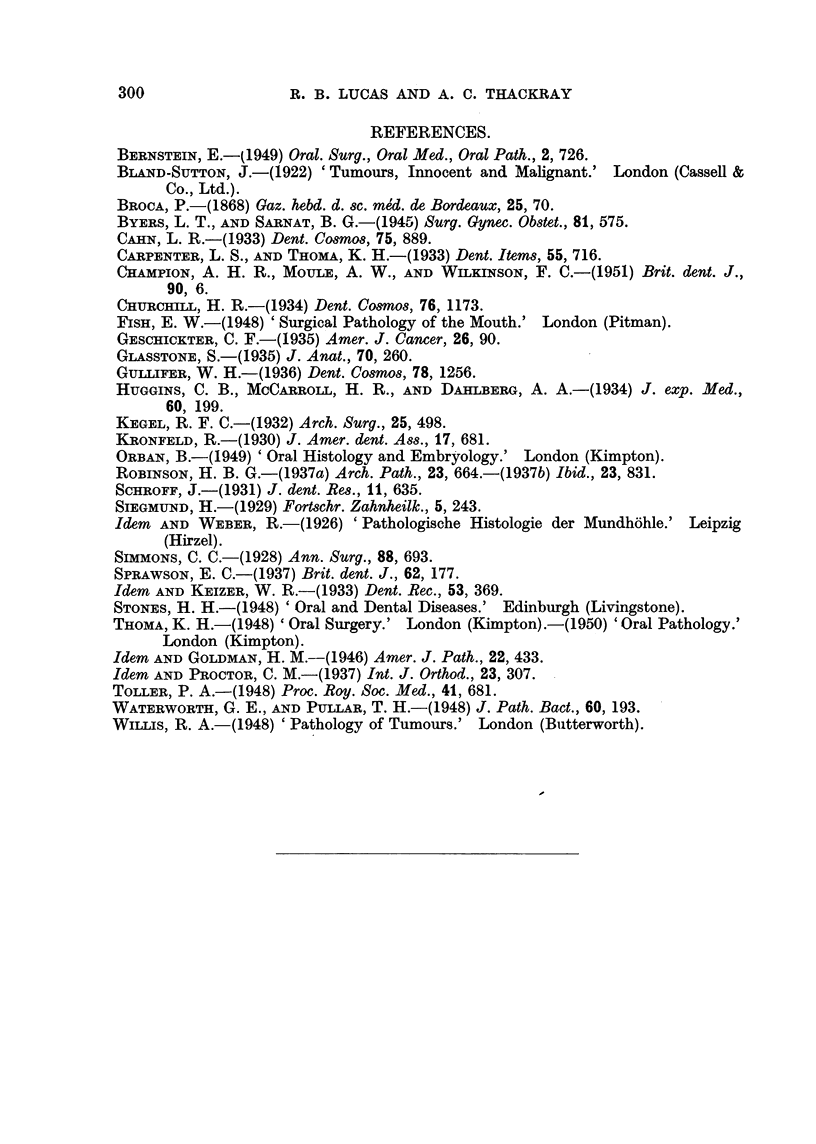# The Histology of Adamantinoma

**DOI:** 10.1038/bjc.1951.30

**Published:** 1951-09

**Authors:** R. B. Lucas, A. C. Thackray

## Abstract

**Images:**


BRITISH JOURNAL OF CANCER

VOL. V SEPTEMBER, 1951 NO. 3

### THE HISTOLOGY OF ADAMANTINOMA.

R. B. LUCAS AND A. C. THACKRAY.

*From the Department of Pathology, The Royal Dental Hospital of London School of Dental Surgery and The Bland-Sutton Institute of Pathology, The Middlesex Hospital, London, W. 1.*

Received for publication August 15, 1951.

THE adamantinoma is one of the less common tumours of the jaw. Its long natural history and marked tendency to recur after inadequate surgery or radiotherapy make an evaluation of methods of treatment difficult. It is important, therefore, that as many detailed cases as possible should be recorded. In this paper the clinical and pathological features of 20 cases are presented and the histogenesis of the tumour is discussed.

### CLINICAL FINDINGS.

These tumours occur most often in early adult life, though cases have been reported in children, and in one case in this series the age of onset was 78. The tumour occurs in the lower jaw much more frequently than in the upper (17 cases and 3 cases respectively in this series), is usually a central lesion and gradually enlarges, in the majority of cases without pain. In the course of time the bone becomes absorbed, and if a cystic loculus is opened infection and discharge follow.

Local recurrence is common following inadequate treatment, but metastases are very rare. A case of pulmonary metastases has been reported by Waterworth and Pullar (1948), who review previous reports.

The natural history of the disease is long ; it is not always easy therefore to assess the efficacy of various methods of treatment. Most authorities consider radical excision of the tumour to be the treatment of choice. The tumour, if small, is removed with a surrounding margin of healthy tissue, though extensive lesions require resection of the jaw (Simmons, 1928 ; Stones, 1948 ; Thoma, 1948). Radiotherapy gives unsatisfactory results.

Table I summarizes the clinical findings in our series.

### HISTOLOGY.

The adamantinoma is an epithelial tumour in which the cellular configuration may take a variety of forms, according to the degree of differentiation of the component cells. Thus the least evolved type of tumour consists of strands of more or less undifferentiated epithelial cells growing in a fibrous stroma, while a tumour which has undergone a greater degree of differentiation consists of follicles or nests of cells approximating in appearance to the developing enamel organ (Fig. 1). The epithelial follicles in such cases consist of an outer layer of columnar cells resembling the similarly situated cells of the enamel organ, while towards the centre the cells become stellate in appearance and cyst formation may occur. On the other hand, the trend of differentiation may be towards a more highly developed type of squamous epithelium, in which case the tumour consists of sheets of squamous cells showing a tendency to cornification, or a basal cell type of growth may be produced.

These tumours may thus present a variety of appearances, from case to case, or in different areas of the same growth, and while attempts at classification on a histological basis are helpful in so far as they indicate the varying degrees of differentiation which can be observed, they are seldom entirely applicable to any given specimen. Thoma (1950) has proposed one such classification, as follows, though he notes that pure types are but infrequently seen:

(1) *Primitive type*, composed of strands of epithelial cells showing little tendency to differentiation, growing in a loose fibrous stroma. When the stroma takes on neoplastic characteristics the tumour is classified as ameloblastofibroma. (2) *Plexiform type*, consisting of strands of cells more highly differentiated than those of the primitive type. (3) *Stellate type*, in which the cells have reached the stellate shape seen in the enamel organ. (4) *Follicular type*, consisting of follicles composed of cells similar to those seen in a rather more advanced stage of development of the tooth germ. At the periphery of the follicles the cells are cylindrical, resembling ameloblasts. (5) *Acanthoma type*. In this type differentiation into squamous cells occurs. (6) *Adeno-ameloblastoma*. The epithelium is arranged in a glandular pattern. (7) *Haemangio-ameloblastoma*. A very rare type of tumour in which haemangiomatous characters are present. (8) *Melano-ameloblastoma*. Also a very rare tumour, in which the epithelial cells contain pigment.

In our series 4 cases were of the primitive type, whilst the remainder were largely follicular or showed mixed appearances. Fig. 2 (Case 5) shows an example of the primitive type of tumour, cords of epithelial cells growing in a fibrous stroma. At some points there is evidence of commencing differentiation. Similar appearances were seen in Cases 6 and 11. In Case 19 the tumour showed a very cellular stroma (Fig. 3) resembling the mesenchyme of the dental pulp, and can be considered as an adamantinofibroma.

In Case 3 (Fig. 4) the growth is composed of solid masses of cells, though in some areas there is differentiation of the epithelium towards a more columnar type. Case 16 (Fig. 5) shows differentiation of a solid type of growth towards squamous epithelium, with areas of cornification.

The more highly differentiated type of tumour is seen in Fig. 6 (Case 5), in which the growth is composed of follicles or nests of epithelial cells with outer columnar layer and inner stellate area.

Microcyst formation, a common occurrence in adamantinoma, was present in 14 cases. Typically, these cysts occur in the area of stellate cells towards the centres of the follicles, but they also form in the stroma. The occurrence of stromal cysts is a point to which very little previous consideration appears to have been given. Siegmund (1929) noted their occurrence in 2 of his cases, but apart from this instance, and a passing reference by Kronfeld (1930) to the same cases, the literature is silent on the matter. Fig. 7 (Case 14) shows microcysts forming in an epithelial mass, and numerous microcysts are also present in the tumour shown in Fig. 8 (Case 1). In the latter case, however, it will be seen that though there is some breakdown within the follicles, the majority of the cysts have formed on the side of the columnar cell layer opposite to that on which stellate cells are situated. Fig. 9 shows three such cysts in higher magnification. A further example of this type of cyst formation can be seen from another field in Case 14 (Fig. 10, 11). In these cases can be seen evidence of the degenerative changes which precede cyst formation. Thus many of the stromal enclaves show a mucinous type of degeneration, the connective tissue becoming very loose and staining pale blue with haematoxylin. In other precystic areas of stroma foam cells are present. Of these 14 cases showing cyst formation, this change occurred in the epithelium only in 6 cases (Cases 2, 4, 7, 9, 17, 18), in both epithelium and stroma in 7 cases (Cases 1, 3, 8, 10, 14, 15, 20), and in the stroma alone in one case (Case 11). Even in the 6 cases showing epithelial cysts only, evidence could be found in 3 cases of incipient degenerative changes in the stroma.

The cause of cyst formation is a matter of conjecture. It is believed to be the result of progressive degeneration in originally solid growths. The solid tumour closely resembles the developing tooth up to the point at which differentiation of adjacent connective-tissue cells into odontoblasts, and subsequently amelogenesis, would occur in the case of the tooth, but at this stage the tumour cannot develop further. Degeneration therefore takes place (Kronfeld, 1930 ; Robinson, 1937*a*). The ensuing accumulation of fluid of relatively high osmotic tension is probably a factor in the progressive enlargement of the microcysts, a mechanism investigated by Toller (1948) in the case of simple dental cysts, and quite possibly a factor also in the case of cyst formation in these tumours.

With regard to cyst formation in the stroma, it appears that again a process of degeneration is at work, and all stages of breakdown can be seen from a slight loosening of the stroma to mucinous-like degeneration and the formation of completely clear cystic spaces. Though similar degeneration leading to cyst formation may also be observed in basal cell carcinomata the phenomenon is not peculiar to tumour growth as such, since the earlier stages of a similar occurrence can be observed not infrequently in the connective tissue underlying the proliferating epithelium of dental cysts. In fact, it is this stromal degeneration in simple cysts which occasioned Churchill (1934) to emphasize that such areas should not be mistaken for degeneration in the stellate reticulum of adamantinomatous follicles.

### HISTOGENESIS.

The teeth develop as compound structures, the enamel being derived from the oral ectoderm, the dentine, cementum and pulp from mesoderm.

At an early stage in embryonic development an extension of the oral epithelium, the dental lamina, grows into the mesenchyme of the jaw. Ten bud-like thickenings appear at intervals on the lamina, the primordia of the enamel organs for the deciduous teeth, and the cells of the mesenchyme under the buds become condensed to form the primordia of the dentine papillae. Further growth of the lamina leads to the production of similar buds for the permanent teeth, one on the lingual side of each deciduous germ, and those for the permanent molars. The tooth buds thus formed continue to proliferate, producing domed structures with indented deep surfaces surmounting the papillae. The epithelium becomes differentiated into a peripheral layer and a central area, the former constituting the enamel epithelium and the latter the stellate reticulum. The enamel epithelium consists of inner and outer divisions. The inner enamel epithelium is that which lines the concavity of the enamel organ, a single layer of tall columnar cells which will produce enamel, while the outer enamel epithelium consists of cuboidal cells covering the convexity of the organ. The central area of the enamel organ, the cells enclosed by the continuous layers of internal and external enamel epithelium, is the stellate reticulum. The cells here have become separated by intercellular fluid and resemble a network of reticular connective tissue. Between the stellate reticulum and the inner enamel epithelium are several layers of flattened cells, the stratum intermedium.

After the germs are formed in the manner described the dental lamina disintegrates, leaving only isolated groups or nests of epithelial cells between the oral epithelium and the enamel organ, the epithelial rests of Serres.

Enamel is formed by the cells of the inner enamel epithelium, though this cannot take place till the cells of the underlying dentine papilla have differentiated to form odontoblasts and produced dentine. Huggins, McCarroll and Dahlberg (1934) have shown by transplantation experiments that enamel is laid down only on dentine, while Glasstone (1935) demonstrated in tissue culture that odontoblasts can differentiate from mesenchymal tissue only in the presence of enamel epithelium. However, Thoma and Goldman (1946) state that dentine formation is independent of ameloblasts. During the course of enamel formation the stellate reticulum undergoes reduction and finally disappears, so that when the enamel is completely developed the ameloblasts and the cells of the outer enamel epithelium have come into contact, to form the reduced enamel epithelium.

Development of the roots takes place under the influence of Hertwig's epithelial root sheath. This structure is formed by the reflection of the two layers of enamel epithelium at the " mouth " of the bell-shaped enamel organ, and the cells of the inner layer induce the differentiation of connective-tissue cells into odontoblasts. When this has occurred and the first layer of dentine has been deposited, the epithelial sheath breaks up. Its remnants persist as the epithelial rests of Malassez.

Thus the oral ectoderm gives rise to a number of structures in the course of development, from any of which, theoretically, an epithelial tumour can originate. The oral epithelium and its derivatives can be diagrammatically represented, thus :

*Enamel organ.*

The striking resemblance of many adamantinomata to the enamel organ led to the earliest of the theories of origin of these tumours. Broca (1868) considered them to be closely related to the enamel organ, and many writers since have held this opinion. On the other hand, Bland-Sutton (1922) thought that the enamel organ could not be the origin of these growths, since the majority of patients were well into adult life when their tumours first appeared. However, Robinson (1937*b*) in his survey of 379 cases found that the tumour was first noticed between the tenth and thirty-fifth years in 70 per cent of cases. Considered together with the fact that these tumours are very slow growing and have a long natural history, it is evident that the majority of growths originate at quite an early age. Robinson (1937*b*) also found that the tumour occurred most frequently in the premolar and molar regions, where supernumerary tooth germs most often occur, and that many cases were associated with a missing tooth. Kegel (1932) and Geschickter (1935) also support the view that the tumour arises from the enamel organ. Byers and Sarnat (1945) believe that the tumour originates from that structure during the first few years of life, though active growth may not become manifest till later.

If these tumours, or at least the majority of them, originated directly from the enamel organ, it might be expected that on occasion enamel would be formed. This is never the case, though Thoma (1950) considers that the homogeneous zone sometimes to be seen between the basal membrane of the columnar cells in the follicular type of adamantinoma and the stroma, which appears yellow with Van Gieson's stain, must be considered as abortive enamel formation, though it is possible that precystic changes in the stroma could account for such appearances. Thus to ascribe the origin of the tumour to the enamel organ directly implies that the tumour must originate at an early stage, prior to amelogenesis. It must then remain dormant, in some cases for a considerable time.

The occurrence of tumours similar in structure to the adamantinoma in the tibia and in the pituitary must also be considered. In the case of the former the resemblance is probably coincidental (Willis, 1948), though as the pituitary is connected embryologically with the stomatodoeum it is not surprising that tumours derived from it can resemble epithelial tumours of the jaws. But no enamel organs occur in the hypophysis.

A tumour similar in some respects to the adamantinoma, which does produce enamel, is the adamantino-odontoma. This is a rare tumour, though it is the type of growth which might be expected to occur more frequently were differentiated enamel organ epithelium a common source of tumour origin.

*Oral mucosa.*

It has been held that the tumour arises from the oral mucosa. Siegmund and Weber (1926) found numbers of cases in which there was a connection between the oral mucosa and the tumour epithelium, but they were of the opinion that the tumour might equally well have grown up to and established connection with the surface epithelium as have originated from it. Cases of this type have also been reported by Gullifer (1936), Robinson (1937*a*), Fish (1948), Bernstein (1949), Champion, Moule and Wilkinson (1951). Some tumours show areas closely resembling basal cell carcinoma ; Sprawson and Keizer (1933) and Sprawson (1937) believe that the adamantinoma is in fact to be considered as of such nature.

In our series there are 5 cases showing connection with the surface epithelium. An example is Case 1 (Fig. 12), the tumour cells being connected with the surface epithelium over a wide area.

The long duration of the adamantinoma, in most cases as a completely central tumour without involvement of the oral epithelium, is, however, evidence against an origin from the oral mucosa.

These tumours resemble rodent ulcers in several respects. Both are related to derivatives of the covering ectoderm, the one to teeth and the other to hair follicles, though discussion continues as to the stage of development of the appendage at which they arise. Do they come from the fully developed appendage, from surface epithelium which is destined to form such appendages, or from epithelial rests ? The growths also resemble each other in their natural history, growing slowly and rarely if ever metastasizing ; their histological appearance, too, is comparable, and intercellular accumulation of fluid in a rodent ulcer may give it a striking resemblance to the more solid type of adamantinoma.

*Cysts of dental origin.*

A number of cases in which adamantinoma has been considered to have originated in the epithelial lining of a dentigerous cyst have been reported (Schroff, 1931 ; Cahn, 1933 ; Carpenter and Thoma, 1933 ; Thoma and Proctor, 1937). These cases lack the exact proof of histological examination before and after development of the tumour, so that the possibility of the cysts having been of adamantinomatous nature from the outset cannot be excluded. Nevertheless, the evidence given does justify as a presumption the origin of tumours, in some cases, from cyst linings.

*Epithelial rests.*

Malassez first demonstrated the presence of epithelial rests about the roots of the teeth and postulated these as the origin of adamantinomata. Kegel (1932) did not think that the rests of Malassez could give rise to these tumours since, as was then thought, Hertwig's sheath, from which the rests are derived, was composed of outer enamel epithelium only. It is now considered, however, that both layers of enamel epithelium participate in its structure (Orban, 1949).

Epithelial rests appear to be the most likely site of origin for the majority of adamantinomata. They are composed of relatively undifferentiated cells which can give rise to any of the appearances found in these tumours, and moreover will produce a central growth. It is not necessary to postulate tumour formation from these rests in early life, with a subsequent interval of dormancy to account for those cases in which the tumour first appears at a comparatively late age. Being present from the formative phase of the dental tissues onwards they can proliferate at any time, and this seems a more satisfactory hypothesis than the dormancy theory.

The rests of Serre, those left by the dental lamina, are situated quite superficially. They are therefore less likely to be the origin of adamantinomata than the rests of Malassez, which are situated more deeply, within bone.

*Nomenclature.*

Most discussions on the subject of adamantinoma refer to the inappropriate terminology in current use ; it is admittedly difficult to find an acceptable nomenclature for these tumours. The old terms " multilocular cyst " and " epithelial odontome " have rightly been abandoned, while " adamantinoma " and " ameloblastoma " are misnomers, since neither is enamel formed nor are the tumour cells ameloblasts. Willis (1948) suggests " carcinoma of the tooth germ residues " as an appropriate title ; certainly it is free from the objections mentioned above. We have, however, adhered to the more familiar though inaccurate eponym for the sake of brevity.

### Case Histories.

Case 1 : *S. D—, female, b. 1854.—1932 :* The patient first reported with a small polyp inside the left cheek. This was removed after X-ray of the jaw had shown nothing abnormal. *1933 :* Pain was now experienced in the region of the left temporo-mandibular joint and a hard, smooth swelling was found on the outer side of the left superior alveolar margin. The left maxilla was removed and radium treatment commenced. *1934 :* Condition satisfactory. No recurrence. *1936 :* Died. *Histology.*—This is a tumour of the stellate type, with plexiform areas. Numerous microcysts are present, both in the epithelium and in the stroma. Some of the latter contain foam cells. The tumour epithelium is connected with that of the surface over quite a large area.

Case 2 : *W. H—, male, b. 1866.—1880 :* " Jaw scraped." Repeated 4 times in 8 or 9 years. *1928 :* Swelling of right lower jaw of 7 weeks' duration. *1929 :* Much increased in size. Cyst opened. Radon collar, 61·6 mc. in 14 tubes for 6 days. *1936 :* Condition very satisfactory. *Histology.*—The tumour is of the typical follicular variety, showing cystic degeneration within the follicles.

Case 3 : *A. G—, female, b. 1880.—1937 :* Tumour of the left mandible $4\frac{1}{2}$ in. in diameter, of many years' duration. Left mandible removed, from symphysis to upper part of ascending ramus. *1951 :* Very well. *Histology.*—Microscopic examination shows large sheets of polygonal cells in a scanty fibrous stroma. At places the continuous sheets break up to some extent, to form a plexiform arrangement. Here the cells tend to take on a stellate appearance towards the centres of the cords and become elongated and columnar at the periphery. There is some cystic degeneration in the stellate areas and also in the stroma.

Case 4 : *M. H—, female, b. 1893.—1910 :* Dentigerous cyst of one year's duration removed. *1924 :* Recurrence of swelling. *1928 :* Swelling had gradually increased in size till there was now a hard tumour of the right mandible. This was excised and the cavity of a multilocular cyst exposed. The contents were removed. *1931 :* The swelling has gradually recurred. There has always been some pain. There is now a large, hard swelling on the right side of the face adherent to the mandible. Posteriorly it contains a cavity about 0·5 cm. in diameter. X-ray shows expansion of the bone and destruction within the swelling. The right half of the mandible was resected. *1951 :* Condition satisfactory. No recurrence. *Histology.*—This tumour is of the follicular type, consisting of fairly regular nests of stellate cells bounded by columnar epithelium. Cyst formation in the central cells is prominent. One area shows a connection between tumour and surface epithelium.

Case 5 : *C. D—, male, b. 1885.—1905 :* Blow in face followed by painful swelling of left cheek, which was opened intrabucally. *1907, 1912, 1914 :* Three intrabuccal operations for recurrence of swelling. *1914 :* Recurrence. Opened from exterior. *1916 :* Recurrence. *September, 1925 :* There has been progressive swelling of left superior maxilla since 1916. The left side of the hard palate is also swollen and bulges down on to the tongue. All teeth have been removed. Antrum incised. 2 × 50 mg. radium tubes inserted. 2 × 50 mg. radium tubes forced up through hard palate. Tubes left in 18 hours. *October, 1925 :* No improvement. *December, 1925 :* Left side of face swollen over superior maxilla. Severe pain. On left side of hard palate is an irregular swelling extending from mid-line to, and including alveolar margin. *January, 1926 :* Alveolar part of superior maxilla and the palatal tumour excised. Radium 75 mg.

for 12 hours. *May, 1926:* Much improved. *1931:* Condition satisfactory. No recurrence. *1937:* Condition satisfactory. *October, 1939:* Swelling of right side of face. The right antrum is expanded and there is a projection into the right labio-alveolar sulcus. There is evidence of growth at the posterior end of the left maxilla. The right side of the palate is pushed down and irregular in outline. Twenty-eight treatments of deep X-rays given over 38 days. Total tumour dose 6003 r. *June, 1940:* Sequestrum in temporo-mandibular region and abscess in temporal fossa. Pain very severe. Sequestrectomy and abscess evacuated. *December, 1940:* Died of broncho-pneumonia, with osteomyelitis and tumour of jaw. *Histology.*—The tumour is composed of strands of comparatively undifferentiated cells in a cellular fibrous matrix.

CASE 6: *C. S—, male, b. 1887.—1923:* A large multilocular cyst was found in the left mandible. The cyst was opened and a wisdom tooth removed. *1924:* A further operation revealed a cavity the size of a walnut in the horizontal ramus. The cyst contained a mass of tissue resembling a villous papilloma. The cyst was enucleated and packed. Untraced since. *Histology.*—The tumour consists of strands and masses of comparatively undifferentiated cells in a fibrous stroma.

CASE 7: *H. B—, male, b. 1900.—May, 1937:* Admitted to hospital with large swelling in $\overline{43|}$ region, of 2 months' duration. Discharge of sero-pus on pressure. No pain. *June, 1937:* Cyst in $\overline{543|}$ region of right mandible removed. *May, 1951:* Patient has been perfectly well since the operation. *Histology.*—The tumour is composed of large masses of epithelium arranged, in places, in follicles. The epithelium is of the stellate type, with the outer layers of the masses and follicles consisting of cuboidal or columnar cells. Microcysts are present, though in comparatively small number, in the epithelium. At one point there is a connection between surface and tumour epithelium.

CASE 8: *M. S—, male, b. 1867.—1927:* Tumour of right mandible of 6 years' duration. Radium treatment commenced, but not completed. *1934:* Now has a large tumour on the right side of the mandible. X-ray examination shows the mandible to be irregularly expanded by multiple cyst formation. Surface application of radium, 13,440 mgh. The following month a surface application of 22,038 mgh. was given. *1935:* Tumour still present though smaller. *1937:* Tumour large. Radium treatment commenced, but terminated by patient before completion. *1939:* Committed suicide. *Histology.*—This is a growth of the follicular type, the cellular masses consisting of stellate cells bounded by columnar epithelium. Numerous cysts are present, mainly

---

FIG. 1.—Developing tooth germs.
A. Stellate reticulum.
B. Dentine papilla.
C. Remains of dental lamina.
D. Oral epithelium.
The germ on the left is the older, showing deposition of enamel and dentine. × 9.

FIG. 2.—A primitive type of tumour. The epithelial component consists of strands of poorly differentiated cells. × 60.

FIG. 3.—Adamantinofibroma. The stroma is very cellular. × 60.

FIG. 4.—A cellular type of tumour. × 170.

FIG. 5.—Another cellular type of tumour showing an area of keratinization. × 175.

FIG. 6.—The typical follicular variety. The follicles are composed of a layer of columnar epithelium surrounding cells of stellate appearance. × 60.

FIG. 7.—Microcyst formation. The cyst formation is due to breakdown of the stellate cells × 75.

FIG. 8.—Microcyst formation. Numerous microcysts are present, mainly in the stroma. × 50.

FIG. 9.—A field from Fig. 8. The microcysts occur on the side of the columnar cell layer opposite to that on which the stellate cells are placed. Small cysts are also present in the stellate area. × 175.

FIG. 10.—Another example of microcyst formation in the stroma. × 25.

FIG. 11.—A field from Fig. 10. × 65.

FIG. 12.—Connection between tumour and surface epithelium. × 175.

Lucas and Thackray.

7

8

9

10

11

12

in the stroma. In some areas cyst formation is not quite complete, and the preceding mucinoid degeneration of the stroma is well seen.

Case 9: *D. W—, female, b. 1905.—1935:* Cyst removed from /4–8 region. No histological examination made. *1940:* The patient complains of dull pain in left mandible. There is a large, fluctuating swelling in /6—→ region. X-ray showed loculated cyst. The cysts were " saucerized " and packed. A plug was later inserted. *1951:* Very well. No recurrence. *Histology.*—Microscopic examination shows follicles of stellate cells bounded by a layer of columnar epithelium in an abundant, quite dense, stroma. The follicles show central cyst formation.

Case 10: *B. M—, female, b. 1892.—1926:* Noticed a lump in the right upper jaw which she thought to be due to ill-fitting dentures. This was treated with " caustic " and disappeared, but recurred within 4 months. It was again similarly treated. *1928:* On admission to hospital an oval area of enlargement of the right maxilla was noted, situated between the alveolar margin of the maxilla and the inner surface of the cheek, the long axis directed antero-posteriorly for about an inch. The area of enlargement and the maxillary antrum were opened up and curetted. A 50-mg. radium tube was placed in the cavity for 24 hours. Untraced since. *Histology.*—The tumour is of the follicular type in most areas, though in some parts it takes the form of fairly large continuous sheets of epithelium. Cystic degeneration is present in the epithelium and in the stroma.

Case 11: *D. D—, female, b. 1905.—1947:* Occasionally felt some stiffness in the left side of the jaw. *1948:* A lump in the jaw. *1949:* The lump has now begun to discharge. On examination there is a hard mass along the inferior border of the mandible in the /3–6 region, extending upwards to the external surface of the bone to the superior border. /4567 had been removed. Left half of mandible resected. *1951:* No recurrence. General condition satisfactory. *Histology.*—The tumour is composed of strands of epithelial cells in a connective-tissue stroma. The cells are for the most part polyhedral or spheroidal, but in areas where the epithelial strands are thin they tend to be flattened. The stroma is quite loose and shows mucoid degeneration, with the formation of microcysts.

Case 12: *E. W—, female, b. 1899.—1928:* Operated on for " abscess of jaw." *1937:* Painful swelling appeared at site of previous operation. X-ray examination showed a multilocular cyst in the 3–/–6 region. The cyst was opened. *1940:* Swelling recurred and cyst again opened and contents removed. *1943:* No recurrence. *1951:* Recurrence, but very small. *Histology.*—The tumour consists of islets of epithelial cells in a plentiful, fairly dense, fibrous stroma. The epithelial islets consist of an outer layer of cubical cells enclosing a central stellate area.

Case 13: *F. B—, female, b. 1887.—1929:* The patient complained of a lump on the right side of the jaw of 10 years' duration, which had been operated on 3 times, 10, 6 and 3 years previously. It had increased in size, painlessly, but rather rapidly since the last operation. On examination there was a large swelling on the right side of the mandible, showing the typical X-ray appearances of adamantinoma. The right side of the mandible was removed. *1951:* Condition satisfactory. *Histology.*—Microscopic examination shows typical epithelial clumps lined by columnar cells and containing stellate cells in the central areas. Cyst formation is well marked. The stroma is quite plentiful and fairly dense, though around some of the epithelial islands it tends to be looser and shows evidence of degeneration.

Case 14: *A. W—, male, b. 1881.—1937:* Three years' history of swelling of the right lower jaw after extraction of 3 teeth. This was considered to be due to a dental cyst, and the jaw was scraped on two occasions. After the first operation the swelling disappeared. On its recurrence a second scraping was performed, but the swelling persisted and increased in size, especially during the last year. There was never any pain. Examination showed a large swelling on the lingual and buccal aspects of the premolar and molar regions. X-ray showed expansion of the jaw by a loculated, cystic area. The mandible was resected. *1950 :* Died from other causes. No recurrence. *Histology.*—The tumour is composed of typical epithelial nests with reticular centres, showing in some areas cystic degeneration. The majority of the cysts seen in this tumour, however, occur in the stroma.

CASE 15 : *E. N—, male, b. 1904.—1945 :* Cyst in right lower jaw, of 6 months' duration. Two teeth and cyst removed. *1947 :* There has been residual swelling in the jaw since removal of the cyst. This has now started to enlarge. X-ray examination shows entire ramus occupied by cyst, up to the coronoid process. Right half of mandible resected. *1950 :* No recurrence. General condition satisfactory. *Histology.*—The tumour shows typical epithelial follicles in a fibrous stroma. Many of these are undergoing central cyst formation, and there are also some cysts in the stroma.

CASE 16 : *D. H—, male, b. 1883.—1937 :* Admitted to hospital for a fracture of the elbow-joint, when a large swelling of the right mandible was noticed. This appeared to have started some 5 years previously, and had been gradually increasing in size. X-ray examination showed the typical appearances of adamantinoma, and local excision of the cyst was undertaken. The patient died a few years later from other causes. *Histology.*—The tumour is composed of masses of oval or pyriform cells showing a tendency towards an alveolar type of arrangement. Areas of keratinization are present. The stroma is dense and shows comparatively few nuclei ; it is hyaline at some points. Necrosis and haemorrhage occur in a number of areas, and involve both epithelium and connective tissue equally.

CASE 17 : *M. K—, female, b. 1905.—1923 :* Tooth removed from left lower jaw ; the socket did not heal properly. *1938 :* Dental cyst removed from same region. *1946 :* Enlargement of the left mandible, painless and not tender. X-ray examination showed a large cyst extending well up into the ramus. *1948 :* Left half of mandible removed. *Histology.*—The tumour consists of epithelial follicles bounded by columnar epithelium. The follicles contain stellate cells and show microcyst formation.

CASE 18 : *H. B—, male, b. 1876.—1927 :* Noticed a swelling in the jaw. This was operated on. *1931 :* Growth recurred. Operation. *1937 :* Recurrence. On examination there was a large, firm mass, 3·5 cm. in diameter, below the angle of the mandible on the left side. Behind this was a smaller mass 1 cm. in diameter. Both were fixed to the deep tissues, but not to the skin. Deep X-rays given over 4 fields in 15 treatments with a total of 5200 r. Second course given 6 months later over 3 fields in 21 treatments. Total dose 6600 r. *1939 :* Mass still present, but patient very well. *1940 :* Condition satisfactory. Mass still present but smaller, and apparently composed of sclerotic bone only (by X-ray). *1946 :* Very well. No recurrence. *Histology.*—The tumour consists of typical epithelial follicles in a fibrous stroma. Microcyst formation is present in the follicles.

CASE 19 : *E. S—, female, b. 1929.—1946 :* Swelling of left side of mandible of 6 weeks' duration. No pain. On examination there is an ovoid area of expansion in the $\overline{|456}$ region. Segment of mandible excised. *1951 :* Very well. No recurrence. *Histology.*—The tumour is composed of islets of cubical epithelial cells in a very cellular stroma.

CASE 20 : *K. C—, male, b. 1927.—1951 :* Noticed a small swelling in the lower jaw 3 years ago. This has become progressively larger and recently it has been painful. On examination there is a swelling 2½ in. in diameter extending from the body of the mandible along the ramus. X-ray showed a loculated cyst, and at operation the cyst was opened and the lining removed. *Histology.*—The tumour consists of areas of stellate cells bounded by a single layer of columnar epithelium. There is cyst formation both in the epithelium and in the stroma.

TABLE I.

| Case. | Sex. | Site. | Age at onset. | Age at main treatment. | Treatment. | Result. |
|---|---|---|---|---|---|---|
| 1 | F. | Maxilla | 78 | 79 | Resection of maxilla and radium | No recurrence after 1 yr. |
| 2 | M. | Mandible | First jaw symptoms at 8 yrs. Tumour appeared 40 yrs. later | 63 | Radon | ,, ,, 7 yrs. |
| 3 | F | ,, | ? | 57 | Resection of mandible | ,, ,, 10 yrs. |
| 4 | F. | ,, | Dentigerous cyst at 17 yrs. Tumour appeared at 31 yrs. | 38 | Ditto | ,, ,, 20 yrs. |
| 5 | M. | Maxilla | 20 | 40<br>54 | Radium<br>X-rays | No further information. |
| 6 | M. | Mandible | 36 | 37 | Enucleation of cyst | ,, ,, |
| 7 | M. | ,, | 37 | 37 | Ditto | No recurrence after 14 yrs. |
| 8 | M. | ,, | 54 | 60<br>67<br>70 | Radium<br>,,<br>,, | Tumour still present. |
| 9 | F. | ,, | 30 | 35 | Saucerization of cyst | No recurrence after 11 yrs. |
| 10 | F. | Maxilla | 34 | 36 | Radium | No further information. |
| 11 | F. | Mandible | 42 | 44 | Resection of mandible | No recurrence after 2 yrs. |
| 12 | F. | ,, | 29 | 29<br>38<br>41 | Enucleation of cyst | Recurrence after 11 yrs. |
| 13 | F. | ,, | 32 | 42 | Resection of mandible | No recurrence after 22 yrs. |
| 14 | M. | ,, | 53 | 56 | Ditto | ,, ,, 13 yrs. |
| 15 | M. | ,, | 41 | 43 | ,, | ,, ,, 3 yrs. |
| 16 | M. | ,, | 49 | 54 | Excision of cyst | No recurrence after some years. |
| 17 | F. | ,, | ? | 41 | Resection of mandible | No further information. |
| 18 | M. | ,, | 51 | 61 | X-ray | No recurrence after 9 yrs. |
| 19 | F. | ,, | 17 | 17 | Resection of mandible | ,, ,, 5 yrs. |
| 20 | M. | ,, | 24 | 24 | Removal of cyst lining | To be followed by excision of mandible. |

SUMMARY.

1. Twenty cases of adamantinoma are described.

2. The histology of the tumour is discussed and the stromal origin of some of the cysts is emphasized.

3. The histogenesis of these tumours is considered. Reasons are given for believing that they originate in the rests of Malassez in most cases.

We are indebted to those surgeons of the Royal Dental Hospital and the Middlesex Hospital whose cases we have studied, and particularly to Mr. D. Greer Walker. Part of the expenses of this study were defrayed by the British Empire Cancer Campaign.

### REFERENCES.

BERNSTEIN, E.—(1949) *Oral. Surg., Oral Med., Oral Path.*, **2**, 726.
BLAND-SUTTON, J.—(1922) 'Tumours, Innocent and Malignant.' London (Cassell & Co., Ltd.).
BROCA, P.—(1868) *Gaz. hebd. d. sc. méd. de Bordeaux*, **25**, 70.
BYERS, L. T., AND SARNAT, B. G.—(1945) *Surg. Gynec. Obstet.*, **81**, 575.
CAHN, L. R.—(1933) *Dent. Cosmos*, **75**, 889.
CARPENTER, L. S., AND THOMA, K. H.—(1933) *Dent. Items*, **55**, 716.
CHAMPION, A. H. R., MOULE, A. W., AND WILKINSON, F. C.—(1951) *Brit. dent. J.*, **90**, 6.
CHURCHILL, H. R.—(1934) *Dent. Cosmos*, **76**, 1173.
FISH, E. W.—(1948) 'Surgical Pathology of the Mouth.' London (Pitman).
GESCHICKTER, C. F.—(1935) *Amer. J. Cancer*, **26**, 90.
GLASSTONE, S.—(1935) *J. Anat.*, **70**, 260.
GULLIFER, W. H.—(1936) *Dent. Cosmos*, **78**, 1256.
HUGGINS, C. B., MCCARROLL, H. R., AND DAHLBERG, A. A.—(1934) *J. exp. Med.*, **60**, 199.
KEGEL, R. F. C.—(1932) *Arch. Surg.*, **25**, 498.
KRONFELD, R.—(1930) *J. Amer. dent. Ass.*, **17**, 681.
ORBAN, B.—(1949) 'Oral Histology and Embryology.' London (Kimpton).
ROBINSON, H. B. G.—(1937*a*) *Arch. Path.*, **23**, 664.—(1937*b*) *Ibid.*, **23**, 831.
SCHROFF, J.—(1931) *J. dent. Res.*, **11**, 635.
SIEGMUND, H.—(1929) *Fortschr. Zahnheilk.*, **5**, 243.
*Idem* AND WEBER, R.—(1926) 'Pathologische Histologie der Mundhöhle.' Leipzig (Hirzel).
SIMMONS, C. C.—(1928) *Ann. Surg.*, **88**, 693.
SPRAWSON, E. C.—(1937) *Brit. dent. J.*, **62**, 177.
*Idem* AND KEIZER, W. R.—(1933) *Dent. Rec.*, **53**, 369.
STONES, H. H.—(1948) 'Oral and Dental Diseases.' Edinburgh (Livingstone).
THOMA, K. H.—(1948) 'Oral Surgery.' London (Kimpton).—(1950) 'Oral Pathology.' London (Kimpton).
*Idem* AND GOLDMAN, H. M.—(1946) *Amer. J. Path.*, **22**, 433.
*Idem* AND PROCTOR, C. M.—(1937) *Int. J. Orthod.*, **23**, 307.
TOLLER, P. A.—(1948) *Proc. Roy. Soc. Med.*, **41**, 681.
WATERWORTH, G. E., AND PULLAR, T. H.—(1948) *J. Path. Bact.*, **60**, 193.
WILLIS, R. A.—(1948) 'Pathology of Tumours.' London (Butterworth).